# Evaluation of the Efficacy of Minimal Invasive Methods Versus Conventional Methods for Caries Removal in Primary Molars

**DOI:** 10.7759/cureus.50803

**Published:** 2023-12-19

**Authors:** Noha M Elfeel, Mohammed M Aboelmaaty, Mohamed H Mostafa, Safaa R El-Sayed

**Affiliations:** 1 Department of Pedodontics and Oral Health, Faculty of Dental Medicine for Girls, Al-Azhar University, Cairo, EGY; 2 Department of Pedodontics and Oral Health, Faculty of Dental Medicine for Boys, Al-Azhar University, Cairo, EGY

**Keywords:** dental pain, conservative dentistry, chemomechanical, diamond bur, ceramic bur, brix 3000®

## Abstract

Aim: Evaluating the efficacy of two “minimal invasive methods” of caries removal (Brix 3000/ceramic bur) in comparison with the conventional method in the management of dental caries in primary molars.

Materials and methods: A sum of 60 teeth from children ranging from four to nine years old of both sexes were selected for this study and randomly assigned to three groups: Group I (Brix 3000), Group II (ceramic bur), and Group III (diamond bur), with 20 teeth per group.

Results: Regarding the efficacy of caries removal, the Brix 3000 group had significantly the lowest efficacy, while the ceramic bur and diamond bur groups were significantly the highest without significant differences between the ceramic and diamond groups. Regarding caries removal time, the Brix 3000 group was significantly the highest, while the diamond bur group was significantly the lowest, and the ceramic bur group was intermediate between them. Regarding pain perception, the Brix 3000 and ceramic bur groups were significantly the lowest, with insignificant differences between them, while the diamond bur group was significantly the highest.

Conclusion: Ceramic bur is an excellent alternative treatment for caries removal in children in comparison with conventional methods and CMCR. Brix 3000®, despite being less painful in comparison with drilling methods, has lower efficacy and efficiency.

## Introduction

Over the past few decades, the treatment of dental caries has changed, moving from “extension for prevention to the prevention of extension.” Various techniques, such as airotor, aluminum oxide, air-abrasion, sono-abrasion, and polymer burs, have been used to remove caries over the years. The main drawback of these materials was the likelihood of loss of sound tooth structure and decreased tactile sensation. Minimally invasive dentistry (MID) is the contemporary medical method of managing dental caries that emphasizes early illness prevention and intervention through the use of caries-risk assessment. By shifting the emphasis away from tooth restoration, the dentist can use less invasive techniques to accomplish maximum intervention [1].

In pursuit of utilizing newer technologies for caries removal, chemomechanical methods have been introduced for the elimination of carious dentin, especially in pediatric or anxious patients. They can identify the infected dentin from the affected dentin, providing pulp protection and allowing for remineralization of the affected dentin [1,2].

More recently, a brand-new papain-based agent called Brix 3000 was released with significant compositional changes. It is a proteolytic enzyme derived from the latex and fruits of green papaya (CaricaPapaya) that acts as a chemical debridant. The remarkable features of this product, according to the manufacturers, are the amount of papain used (3,000 U/mg in a 10% concentration) and its bioencapsulation by EBE technology, which provides the gel with an optimal pH for releasing the enzymes at the point of employing its proteolysis effect on the collagen. Furthermore, it has resistance to storage even under undesirable circumstances, does not require cold-chain conservation, greater antibacterial and antifungal, with an improved antiseptic effect on tissues. It also has a dermatological certificate confirming the product’s safety for the mouth, skin, or eyes, indicating that it does not trigger any reactions when it encounters healthy tissue. This explains the ability of this product to remove compromised tissue more quickly and without damage or pulp cytotoxicity [3].

Another self-limiting concept in the mechanical removal of caries was a bur that was made of a special alumina-based ceramic with stabilized zirconia. Ceramic burs can be highly efficient in removing carious dentin with little damage to the structural integrity of the tooth. They are available in four ISO (International Organization for Standardization) sizes: 012, 014, 018, and 023. Ceramic burs, like traditional carbide burs, are utilized in a slow-running handpiece at a speed of 1,000 to 1,500 RPM. According to the manufacturer, the benefits of ceramic burs for dentinal caries excavation include their smooth, pleasant operation, better tactile feeling, optimal cutting effectiveness, and lack of corrosion. Because of this, ceramic burs, as opposed to traditional burs, are appropriate for minimally invasive caries removal, which involves cutting off a small portion of dentinal tubules to reduce sensation-stimulated pain [4].

## Materials and methods

Study design and ethical approval

This secondary care-based, three-arm, parallel group, patient-randomized controlled trial was carried out at the outpatient clinic of the Pedodontics Department, Faculty of Dental Medicine for Girls, Al-Azhar University. Research Ethics Committee approval with code (REC-PE-23-18; dated September 2023) was obtained from the Faculty of Dental Medicine for Girls, AlAzhar University.

Informed consent

Full details of procedures, possible discomfort, and benefits of this study were emphasized to the parents of the children, and signed informed consent for treatment was acquired. Prior to their inclusion in the study, children were also told about the study's purpose in language appropriate for their age.

Sample size calculation and statistical power of the clinical part

The sample size was calculated based on the results of Matar et al. [5]. According to this study, the probability of score 0 among controls is 52.9 in scores 1 and 2 (17.6%), scores 2 and 4 (5.9%), and score 5 (0%). The estimated probability of score 0 among interventions is 85.0% in score 1 (5%), score 2 and 3 (4%), and score 5 (2%), with a 0.65 effect size when the probability (power) is 0.8. The Type I error probability is 0.05. The minimal accepted sample size is 16 per group. It is increased to 20 to compensate for a 20% dropout.

Subject selection

A total of 60 children, ranging from four to nine years old, of both sexes, were invited to participate in the study after meeting the following inclusion criteria: having one primary molar with a cavitated carious lesion, reaching the dentin with ICDAS 4 or 5. All children should be medically free with the lack of clinical evidence of pulpitis and pulpal degeneration (spontaneous pain, pain on percussion, history of swelling, sinus tract) [6].

Clinical examination

Before starting the procedure, comprehensive medical and dental histories were taken, followed by clinical and radiographic examinations. Patient information was gathered and documented in the patient examination chart.

Study groups

Sixty teeth were haphazardly divided into three groups (Group I, Group II, and Group III), 20 teeth for each group, and one primary molar in each patient was treated.

Clinical procedures

Anesthesia was not administered prior to the procedure for the purpose of objectively assessing pain perception [2]. The patient was advised that he had the choice to be anesthetized if he was experiencing pain or discomfort. The rubber dam was not utilized to prevent any discomfort that could be caused by clamp placement since treatment was initiated without anesthesia. So, each tooth was partially isolated using a saliva ejector and cotton roll instead. The time was calculated by a stopwatch to start when the method of caries removal begins until the end of the procedure after the complete removal of the carious lesion. After confirmation of caries removal, all cavities were conditioned by (KetacTM conditioner (3M ESPE, Germany) for 10 seconds, rinsed, and restored with EQUIA Forte HT Fil (GC Corp., Tokyo, Japan), then coated with the light-cure coat (GC Corporation, Tokyo, Japan).

Group I

Brix 3000 (S.R.L., Argentina) was applied to the cavitated carious lesion and, according to the manufacturer's instructions, left on for two minutes. When the gel turned hazy, it was carefully scraped away along with the softened carious dentine using a sharp spoon excavator without utilizing any force. When required, the application was repeated until all the infected dentine was eliminated. The cavity was then rinsed with water [2].

Group II

Ceramic burs (CeraBur, K1SM, Komet Brasseler; Lemgo, Germany) were used with a low-speed hand piece with sizes that were suitable for occlusal caries based on the selection criteria. Without using a water coolant, carious tissue was excavated in circular movements from the cavity's center to the cavity's periphery from the occlusal aspect. After hard dentin was recognized, the caries excavation was ended [4].

Group III

Diamond bur (Many, Japan) was used with a high-speed handpiece and air-water spray to remove caries.

Evaluation of clinical procedures

Evaluation of Caries Excavation Time (Efficiency)

The time needed for the elimination of caries was measured in minutes, beginning with the handpiece, or using a spoon excavator and ending with the final probing for dentin hardness [7].

Evaluation of Caries Removal Efficacy

After complete excavation, the caries removal was confirmed by tactile and visual methods in addition to caries detector dye. The efficacy was graded from 0 to 5 according to the degree of caries removal and was evaluated using the caries removal efficiency scoring system as in Table 1 [4].

**Table 1:** Scoring criteria for the assessment of caries removal efficacy

Score	Criteria
0	Caries completely removed
1	Caries present in the base of the cavity preparation
2	Caries present in the base and/or in one wall of the cavity preparation
3	Caries present in the base and/or two walls of the cavity preparation
4	Caries present in the base and/or more than two walls of the cavity preparation
5	Caries present in the base, walls, and margins of the cavity preparation

Evaluation of Pain and Patient Comfort

**Figure 1:**
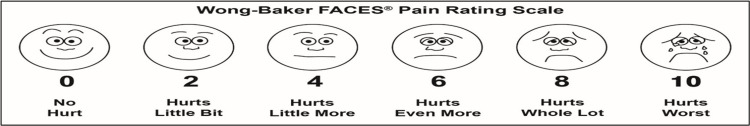
Wong Baker faces pain rating scale.

The objective assessment of pain was conducted using the FLACC scale (face, leg, activity, cry, and consolability scale). Subjectively, the Wong Baker Faces Pain Rating Scale was used, in which the child was asked to rate the pain and discomfort on a 6-point scale, with a smiling child at one end and a tearful child at the other (Figure 1). The selected face was taken as the pain score [5]. 

Statistical analysis

A commercially available software program (SPSS 19; IBM Corp., Armonk, NY, USA) was employed for statistical analysis. The chi-square test was utilized for comparing qualitative data expressed as a number and a percentage. To compare parametric quantitative data, the independent t test was used.

## Results

Caries removal time (efficiency)

Comparison between different groups was carried out using One Way ANOVA test, which revealed that Brix 3000 was significantly the highest regarding the time taken for caries removal (12.08 ± 2.47), then Ceramic bur (8.01 ± 1.58), while Diamond bur (6.28 ± 2.32) was significantly the lowest as P = 0.0001.

Caries removal efficacy results

The frequency and percentages of different scores among all groups regarding caries removal efficacy were presented in Table 2.

**Table 2:** Descriptive statistics of caries removal efficacy and comparison between groups among different scores (chi-square test). N: count, %: percentage *Significant difference as P<0.05.

Score	Brix 3000		Ceramic bur		Diamond bur		
	N	%	N	%	N	%	P value
Score 0	7	33.33%	17	86.67%	15	73.33%	0.0007*
Score 1	8	40.00%	3	13.33%	5	26.67%	0.38
Score 2	5	26.67%	0	0.00%	0	0.00%	0.01*
Score 3	0	0.00%	0	0.00%	0	0.00%	-------
Score 4	0	0.00%	0	0.00%	0	0.00%	-------
Score 5	0	0.00%	0	0.00%	0	0.00%	-------
P value	0.38		<0.0001*		0.003*		

Pain perception results

**Figure 2:**
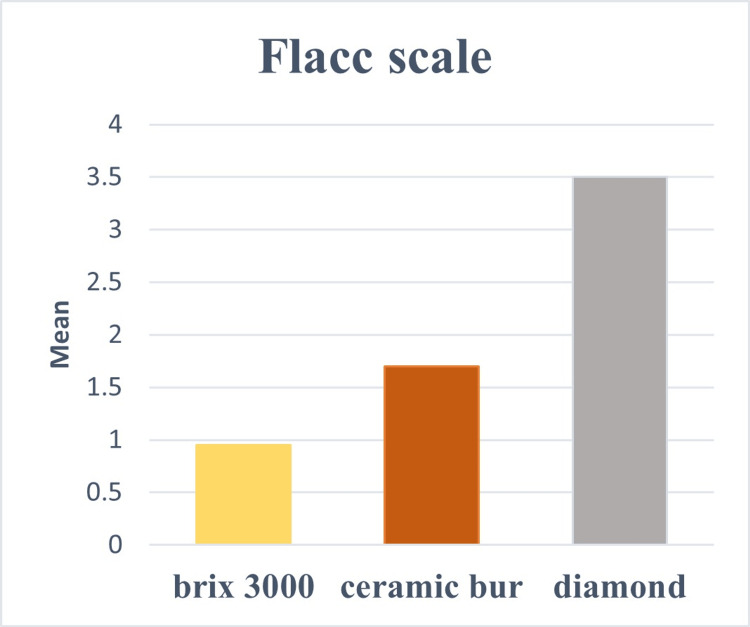
Bar chart representing Flacc scale in all groups.

**Figure 3:**
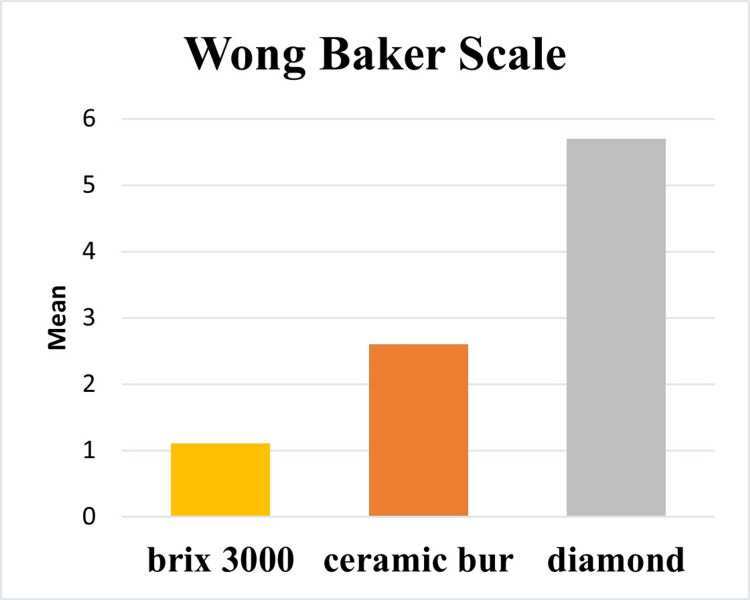
Bar chart representing Wong Baker scale in all groups

Results of pain perception in all groups, including the objective pain scale (Flacc scale), were represented in Table 3 and Figure 2 and subjective pain scale results (Wong Baker pain rating scale) were represented in Table 3 and Figure 3.

**Table 3:** Descriptive statistics of Flacc scale and Wong Baker scale in all groups (chi-square test). Min: minimum, Max: maximum, Med: median, M: mean, SD: standard deviation *Significant difference as P<0.05 Means with different superscript letters were significantly different as P<0.05. Means with the same superscript letters were insignificantly different as P>0.05.

		Min	Max	Med	M	SD	P value
Flacc scale	Brix 3000	0.00	7.00	0.00	0.95 a	1.79	0.006*
	Ceramic bur	0.00	8.00	0.00	1.7 ab	2.43	
	Diamond	0.00	7.00	4.00	3.5 b	2.70	
Wong baker scale	Brix 3000	0.00	2.00	2.00	1.1 a	1.02	<0.0001*
	Ceramic bur	0.00	8.00	2.00	2.6 a	2.35	
	Diamond	2.00	10.00	5.00	5.7 b	3.06	

## Discussion

The conventional method of removing caries is excavation using traditional burs. Being nonselective, they remove both infected and sound dental tissues, causing detrimental biological consequences for dental pulp tissues. It triggers anxiety, fear, and pain and often requires a local anesthetic injection, which compromises the acceptance of dental treatment by children. To overcome these issues, advanced conservative caries removal methods have evolved, only two of them (CMCR) and ceramic bur were the focal points in the present study [8].

Regarding caries efficiency results, diamond bur proved to be the least time-consuming. The reason could be due to the high rotational speed (>20,000 rpm) of the diamond bur with the frictional force presenting a negative rake angle, resulting in grinding the tooth surface rather than cutting the tooth structure during cavity preparation [9,10]. The Brix 3000 group proved to be the most time-consuming, which may be due to the lesion consistency (soft, medium, or hard), which required multiple applications of Brix 3000 gel to decompose the infected dentine, mostly two to three times per case, followed by mechanical excavation, also the need in some cases to use conventional drills to get access to the lesion in the presence of undermined enamel, while ceramic bur is composed of alumina-yttria ceramic, which in general has excellent wear resistance and cutting ability, which makes it faster in caries removal compared with CMCR [2].

That was in harmony with previous studies that found a significant difference between CMCR and mechanical methods regarding caries removal time [9,10]. In disagreement with this result, another study found no significant difference in the operating time between CMCR and conventional methods [11].

Caries removal efficacy results showed that Brix 3000 was significantly the lowest; in 33.33% of cases where the chemo-mechanical method was used, complete removal of the caries lesions was achieved, while in the remaining 67%, the carious lesion was partially removed. In most cases, it tends to leave the firm, leathery, carious dentine in the base and wall of the cavity. That was in harmony with a previous study that reported that the efficacy of caries removal by the CMCR system only showed a success rate of only 36% of treated cases, indicating that it did not remove the caries effectively and therefore cannot replace the rotary instruments [12].

Although there was no significant difference between the diamond and ceramic bur groups, ceramic bur scored the highest in complete caries removal (score 0) (86.67%), while diamond bur was (73.33%). Ceramic bur has the advantage of being minimally invasive and less painful, making it a better choice for caries removal in children. That was in accordance with another study that found no significant difference between ceramic and conventional burs regarding caries removal efficacy [13]. Contradictory to this study, a previous study showed significantly higher residual caries at the cavity floor with ceramic burs than conventional burs [14].

Regarding pain scale results, Brix 3000 and ceramic bur were significantly the lowest, with insignificant differences between them, while diamond bur was significantly the highest. This is maybe due to the fact that Brix 3000 contains the papain enzyme that interacts with exposed collagen through the dissolution of dentin minerals through bacteria, making the infected dentin softer allowing its removal with non-cutting instruments easily without local anesthesia and burs. Besides the fact that caries removal using CMCR takes place in the absence of vibration and noise, in addition to the characteristics of heat insulation, this resulted in maximum preservation of the healthy tooth structure and minimized the use of local anesthesia [15].

Ceramic burs also have the advantage of being a minimally invasive method that selectively removes the infected dentine. Hence, less cutting of dentinal tubules and decreasing sensitivity. Moreover, the use of ceramic bur provides better tactile control, as when it reaches sound dentine, some resistance, and vibration are felt, which is suggestive of no further removal of caries. That agreed with a previous study that found Brix 3000 was more comfortable than a ceramic bur, which minimized the demand for local anesthesia along with drill use. There is little difference in the acceptance of the patients between the two groups after treatment [16].

On the other hand, the conventional method of removing cavities with drills caused unneeded removal of the sound or even affected dentin, which hindered the process of remineralization and exposed more dentinal tubules, leading to more pain stimulation in addition to the noise, vibration, and heat generated during tooth preparation. Many patients reported this to be an unpleasant and uncomfortable sensation, and in many of these situations, local anesthetics were used to control the pain. That agreed with the findings of an earlier study [7]. This contradicted a previous study, which found no difference in anxiety levels between the CMCR and conventional groups during and after treatment [17].

These results showed that ceramic burs combine efficacy, being able to remove caries and get access to the lesion without the help of conventional burs, and being less painful and minimally invasive, removing only infected dentine. Ceramic bur is also cost-effective compared to chemomechanical caries removal systems. It is sterilizable, and according to its manufacturer, it is service life is three times longer than that of tungsten carbide burs, making it a better choice than its precursors (smart burs) as it is abraded easily, demanding an indefinite number of burs that can be used per case. That was in accordance with a previous study [18].

## Conclusions

Ceramic bur is an excellent substitutive treatment for caries removal in children as it blends efficacy, efficiency, and comfort in comparison with conventional methods. Moreover, it is minimally invasive providing maximum preservation of the tooth structure, in addition to being cost-effective in comparison with the CMCR group (Brix 3000).

Brix 3000®, despite being minimally invasive and less painful in comparison with drilling methods, has a lower efficacy and efficiency for caries removal. It can be used as an alternative to caries removal in young and fearful children who have high anxiety levels regarding vibrations and noise.
